# A bootstrap based analysis pipeline for efficient classification of phylogenetically related animal miRNAs

**DOI:** 10.1186/1471-2164-8-66

**Published:** 2007-03-06

**Authors:** Yong Huang, Xun Gu

**Affiliations:** 1 Department of Genetics, Development, and Cell Biology, Center for Bioinformatics and Biological Statistics, Iowa State University, Ames, IA 50011, USA

## Abstract

**Background:**

Phylogenetically related miRNAs (miRNA families) convey important information of the function and evolution of miRNAs. Due to the special sequence features of miRNAs, pair-wise sequence identity between miRNA precursors alone is often inadequate for unequivocally judging the phylogenetic relationships between miRNAs. Most of the current methods for miRNA classification rely heavily on manual inspection and lack measurements of the reliability of the results.

**Results:**

In this study, we designed an analysis pipeline (the Phylogeny-Bootstrap-Cluster (PBC) pipeline) to identify miRNA families based on branch stability in the bootstrap trees derived from overlapping genome-wide miRNA sequence sets. We tested the PBC analysis pipeline with the miRNAs from six animal species, *H. sapiens, M. musculus, G. gallus, D. rerio, D. melanogaster*, and *C. elegans*. The resulting classification was compared with the miRNA families defined in miRBase. The two classifications were largely consistent.

**Conclusion:**

The PBC analysis pipeline is an efficient method for classifying large numbers of heterogeneous miRNA sequences. It requires minimum human involvement and provides measurements of the reliability of the classification results.

## Background

MicroRNAs (miRNAs) are small (~22 nucleotides) non-coding RNAs that have the ability to repress the expression of target genes post-transcriptionally. Since the discovery of the first two miRNA genes, *lin-4 *[1,2] and *let-7 *[3,4], much has been learned about the structure, biogenesis and function of miRNAs [5-7]. A growing number of miRNAs have been found in animals, plants and viruses [8,9]. The "miRBase" database [10] currently hosts 4039 miRNAs from 45 species (Release 8.2).

Studies in plants [11], animals [12,13], and viruses [8] have shown that the innovation of miRNAs is an ongoing process [14,15], which indicates that most miRNAs are of different evolutionary origins. Some miRNAs, however, were also populated through local or genome-wide duplications [14,16], and formed miRNA families. As the phylogenetically related miRNAs convey important information about the function and evolution of miRNAs, a reliable classification of miRNA families is indispensable for miRNA studies.

Some work has been done in classifying miRNA families. The nomenclature system for miRNAs by "miRBase" [17] can be view as one of the earliest classifications. It classified miRNAs with similar mature forms together and assigned them the same id numbers. In recent releases of miRBase, a "miRNA family (miFam)" feature was present, which clustered similar miRNA precursors together based on computational analysis and manual inspection. In a recent report by Hertel et al [14], the phylogenetic distribution of miRNAs in 30 species was systematically studied. In that study, potential miRNAs in the species were identified with BLAST [18] and profile based methods [19]. The phylogenetic analysis was based on pair-wise sequence identity between precursors, and the significance of the sequence identity cutoff was evaluated with a *z*-score.

These studies have revealed important phylogenetic information about miRNAs. However, there are some underlying problems with the current methods. First, the mature forms of miRNAs were often used as the classification criteria in the methods, intentionally or not. This can decrease the sensitivity of finding paralogous miRNAs, as the mature part of a duplicated copy of a miRNA is not necessarily under strong selective pressure. Meanwhile, due to the short length of the mature forms, false classification caused by convergent evolution is very likely to happen. Second, since a fixed sequence identity cutoff value alone is inadequate to classify the miRNAs (See additional file 1: Classification by BLAST), most of the current methods relied on manual inspection in deciding the families. This introduces a heavy human factor in the classification process. Third, most of the current methods do not have a measurement of the reliability of the classification results. The *z*-score used by Hertel et al [14] was no exception, because the *z*-score was actually a measurement of the significance of a fixed sequence identity cutoff value (a BLAST-like e-value could be directly derived from a z-score).

In this study, we designed a bootstrap based analysis pipeline (the Phylogeny-Bootstrap-Cluster (PBC) pipeline) to identify phylogenetically related miRNAs. In our method, the families are identified based on branch stability in the bootstrap trees derived from overlapping input sets of genome-wide miRNA precursor sequences. This approach is similar to the "nodal stability" approach [20] that has been used in phylogenetic tree inference. The difference is that we vary the input data set rather than multiple sequence alignment parameters. A "Vote" algorithm was designed to automate the process of identifying and evaluating potential families. The human involvement in the classification process was minimized, and the reliability of each family was evaluated by its supporting levels from the bootstrap trees. We tested the PBC analysis pipeline with the miRNAs from the six animal species, *H. sapiens, M. musculus, G. gallus, D. rerio, D. melanogaster*, and *C. elegans*. The resulting classification was compared with the miRNA families defined by miRBase. While the classifications were largely consistent, our reliability measurement showed that a few new families can be supported and several families in miRBase may not be supported. The PBC analysis pipeline offers an efficient and objective method for classifying large amount of miRNAs.

## Results

### Algorithm

#### The phylogeny-bootstrap-clustering (PBC) analysis pipeline

This method is base on the branch stability in the bootstrap trees derived from overlapping input sets of genome-wide miRNA precursor sequences. For a set of miRNA sequences, we can always perform a multiple sequence alignment (MSA) and build a bootstrap tree from the alignment result. Due to the imperfection of MSA, it is almost certain that some false classifications will happen. However, the true classifications are generally more robust to variations in input sequences or alignment parameters of MSA than the false classifications [20]. Based on this principle, we can add additional sequences to the original input sequence set of the MSA, and generate a new bootstrap tree. The true families should be more stable and are more likely to be intact under a branch of high bootstrap value in the new tree, while the falsely classified families are more likely to be broken up in the new tree. If multiple new trees are built with different additional sequences, the likelihood for a falsely classified family to be intact in all the new trees decreases geometrically. In practice, for efficient classification of large amount of miRNAs, the original input sequences and the additional input sequences can be genome-wide collections of miRNAs.

**Figure 1:**
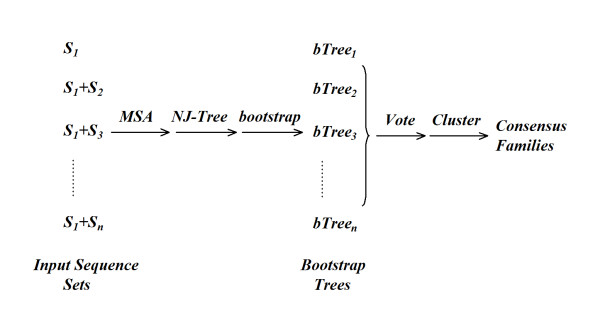
The flowchart of the PBC analysis pipeline.

Suppose we have *n *species whose miRNA information is available. Let *S*_*i *_denote the set of miRNA precursor sequences in species *i*, and let *S*_*i*_+*S*_*j *_denote the union of *S*_*i *_and *S*_*j*_. In the PBC analysis pipeline, we use the sequence sets (*S*_1_, *S*_1_+*S*_2_, ..., *S*_1_+*S*_*n*_) as the input, where *S*_1 _is the original input sequence set and *S*_2_, ..., *S*_*n *_are different additional sequences. MSA, neighbor-joining (NJ) tree building and bootstrapping are carried out for each input sequence set, and *n *corresponding bootstrap trees are built (*bTree*_1_, ... *bTree*_*n*_) (see Figure 1). In these input sets, the *S*_1 _set of sequences appear in all the input sequence sets. The addition of other sequence sets (*S*_2_, ..., *S*_*n*_) to *S*_1 _introduces variations to the input of MSA. From the bootstrap trees (*bTree*_1_, ... *bTree*_*n*_), we identify the branches with bootstrap values above the family defining cutoff values and denote such branching nodes as the "family defining nodes." If all the *S*_1 _leaves under such a branch are also clustered together under a "family defining node" in all the other trees, these leaves form a consensus family. The detailed procedure of this step is capsulated in the "Vote" algorithm described in the next section. A consensus family may contain sequences from several species, but only the classification of the *S*_1 _sequences is confirmed in this step. The reliability of the classification of a group of *S*_1 _sequences in a family can be measured as the average of the bootstrap values of their best common ancestors in the *n *input trees.

Once the *S*_1 _sequences are classified, for an individual sequence *x *from (*S*_2_, ..., *S*_*n*_), we can carry out another PBC analysis with the input sequence sets as (*S*_1_+*x*, *S*_1_+*x *+*S*_2_, ..., *S*_1_+*x *+*S*_*n*_; removing redundant *x *wherever it occurs) to classify *x *with its phylogenetically related miRNAs in *S*_1_. However, for genome-wide sets of sequences, this is computationally prohibitive (as each run of the PBC analysis take hours). Instead of confirming individual sequence, in practice, we formulate a confirmation set of sequences *C *which is composed of the sequences from (*S*_2_, ..., *S*_*n*_) whose phylogenetic relationship with miRNAs in *S*_1 _is of interest. We also remove the most obviously *S*_1_-specific miRNAs from *S*_1 _according to the result of the first round, these include the miRNAs in singleton families in the first round and the miRNAs in tandem clusters. A new round of PBC analysis is then carried out using the input set of (*S*_1_+*C*, *S*_1_+*C *+*S*_2_, ..., *S*_1_+*C *+*S*_*n*_) (removing redundant sequences wherever they occur; *S*_1_-specific sequences removed from *S*_1_). Those sequences in the confirmation set *C *whose classifications are confirmed in the new round are patched back to the consensus families (of the first round). The resulting consensus families form a classification of miRNAs that are phylogenetically related to *S*_1 _miRNAs. In other words, *S*_1 _miRNAs are the index for this classification. Phylogenetic relations among *S*_2_, ..., *S*_*n *_sequences that are not related to any *S*_1 _sequences are not covered in this classification, but such relations can be examined in the same way by using a different set of sequences as index.

#### The "Vote" algorithm

The "Vote" algorithm is the kernel of the PBC analysis pipeline. The input for the "Vote" algorithm includes the set of bootstrap trees (*bTree*_1_, ... *bTree*_*n*_), the "family defining bootstrap cutoff values" which are derived from well studied known families and are tree specific, and a "evaluation bootstrap cutoff value" which is used to evaluate families defined in other trees. Usually, the "family defining cutoff values" are set to be greater than the "evaluation cutoff value".

**Figure 2:**
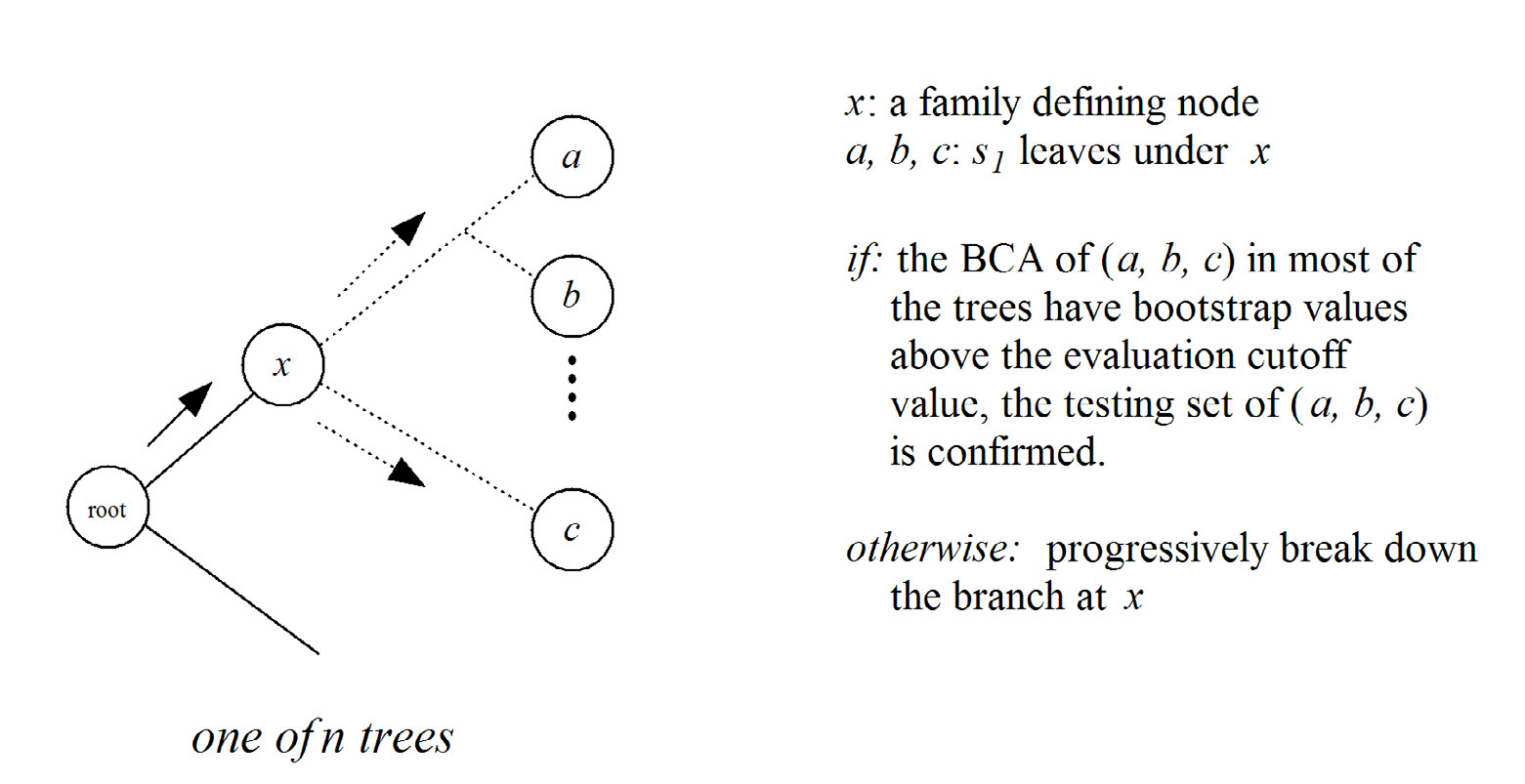
The "Vote" algorithm.

The "Vote" algorithm (see Figure 2) is designed to identify families from the bootstrap trees and get measurements of reliability of the identified families. Suppose the *S*_1 _sequences form the index, as is in the case of the first round of PBC. First, for each tree and from root down, the branches with bootstrap value above (>=) the family defining cutoff values are sought. The search will not go inside a branch if the top node of the branch already has a bootstrap value above the cutoff. The top nodes of these branches are the "family defining nodes." For each identified branch, the *S*_1 _leaves in the branch form a testing set. Second, for each testing set, we can obtain the bootstrap values of their Best Common Ancestor (BCA) in the input trees. The BCA of a set of nodes in a bootstrap tree is defined to be the common ancestor with the highest bootstrap value among all the common ancestors of the set of nodes (See additional file 2: Best common ancestor (BCA)). Third, the reliability of a testing set is evaluated by its BCA bootstrap values in the input trees. If the BCA bootstrap value is above the evaluation cutoff value in a tree, the testing set is regarded as supported by that tree. If a testing set is supported by enough input trees, it is deemed as confirmed (this is where the name "Vote" comes from). In practice, we request a testing set to be supported by *n-1 *trees, allowing one exception. If a testing set is not confirmed, it is progressively broken down until all its subsets are confirmed. Finally, all the confirmed sets are clustered into consensus families using a single linkage clustering approach [21].

### Testing

Classification of miRNAs from six animal species using the PBC analysis pipeline

The input for the testing case were the miRNAs from six animal species, *H. sapiens, M. musculus, G. gallus, D. rerio, D. melanogaster*, and *C. elegans*. These miRNAs cover most of the experimentally identified miRNAs. *S*_1 _was set to be the human miRNAs and *S*_2_, ..., *S*_6 _were the miRNAs from the other five species. MSA was carried out with CLUSTALW 1.83 [22]. Neighbor-joining trees building and bootstrapping were carried out with Mega 3.1 [23] using 1000 bootstrap replications and p-distance substitution model. The family defining bootstrap cutoff values are tree-specific, and are set to be the smallest bootstrap value of the reference miRNA families (let7, mir-124, mir-17 and mir-1, See additional file 3: Reference miRNA families) in each input tree. These reference families are well established in the literature. The actual cutoff values were 84% for the "hsa" tree, 90% for the "hsa-mmu" tree, 91% for the "hsa-gga" tree, 78% for the "hsa-dre" tree, 75% for the "hsa-dme" tree and 82% for the "hsa-cel" tree. The evaluation cutoff value is set to be 50%.

After the first round of the PBC analysis, we examined the composition of the consensus families. For human miRNAs with same id numbers, only 2 are separated in the consensus families, namely mir-92/mir-92b and mir-449/mir-449b, showing that most of the miRNA families are robust to the variation in the input of the PBC pipeline. We further examined the alignments for mir-92/92b and mir-449/449b. In both cases, the mature sequences had high sequence identities while the hairpin sequences had low identities outside the mature parts. The miRNAs in these cases are separated into different families, as they may be the results of convergent evolution.

The confirmation set of sequences were deduced from the consensus families of the first round of PBC. The confirmation set includes the miRNAs from the other species that were not classified with their human counterparts (having the same id numbers). There were 1 from mouse, 0 from chicken, 2 from fish, 5 from fly, and 3 from worm (including cel-lin-4 and cel-lsy-6). The confirmation set also includes the non-human miRNAs that were classified in a family with none of its human counterparts. There were 13 from mouse, 1 from chicken, 12 from fish (selected 3 from the dre-mir-430b miRNA cluster), 20 from fly, and 16 from worm. A second round of PBC analysis was carried out. 42 of the miRNAs in the confirmation set were confirmed and were patched back to the consensus families and the unconfirmed miRNAs were kept out of the families. The resulting consensus families formed the classification of phylogenetically related miRNAs with the human miRNAs as the index. The full list can be found in additional file 4: Full list of the families with human member. The supporting levels of the human miRNAs in each family can be found in additional file 5: Supporting levels of the families.

#### The phylogenetic distribution of miRNAs

Based on the family composition, we categorized the miRNA families in the testing case into categories I-IV. Category I contains miRNA families that have miRNAs from both mammals and invertebrates. In addition, we demand that all the Category I families should have chicken or fish miRNAs. Category II contains miRNA families that have miRNAs from both mammals and non-mammal vertebrates. Category III contains miRNA families that have miRNAs from mammals only. Category IV contains the miRNA families that have no mammal miRNAs. The distribution is summarized in Table 1. An abbreviated list of the human miRNAs of the families is shown in Table 2. It should be noted that worm or fly miRNAs are present in eight families in category III. These families were put in category III because there were no chicken or fish miRNAs in these families. These cases are likely to be the result of convergent evolution or incomplete miRNA discovery in chicken and fish. Most of the human miRNAs in these eight families are from a recent publication [24].

Although the discovery of miRNAs is not comprehensive in species like chicken and fish, the phylogenetic distribution of the miRNA families still shows interesting trends. The majority of the human miRNAs are conserved only in vertebrates. Among the human miRNAs, 47 are in category I, 146 are in category II, and 269 are in category III. This shows that more than half of the human miRNAs are conserved in mammals only, and the majority of the rest are vertebrate only. Only a small portion of human miRNAs have invertebrate homologues. Mouse miRNAs display a similar distribution. For the invertebrate miRNAs, the portion of miRNAs that are conserved in vertebrates is also very small, 22/78 in *D. melanogaster *and 6/114 in *C. elegans*. Only a small number of miRNAs, the 15 miRNA families in category I, are conserved between invertebrates and mammals. However, more than half of all the miRNAs with known function are in these families. This shows a high correlation of sequence conservation and functional importance among the miRNAs. The discovery of miRNAs is still ongoing, so more data and analysis will be needed to generate a quantitatively more detailed phylogenetic distribution of miRNAs.

#### Comparison with the miFam classification

Since Release 8.1, a miFam (miRNA family) feature which provides family classification information of miRNA hairpin sequences has been available in miRBase. We compared our classification results with the miFam classification in Release 8.2. Not considering the families with one or less human miRNA or the families without non-human miRNAs, our comparison showed that 122 out of the 172 comparable PBC families are the same in miFam. While most of the miRNA families are consistent, we examined the cases where the classification was different (summarized in Table 3). Most of these differences involve additional merging or separation of the families between the two classifications. For the families that are merged in the PBC classification, the multiple sequence alignments of the hairpin sequences are generally acceptable, but the alignments of the mature sequence are not necessarily strong. One example is the mir-134/mir-412 case (See additional file 6: Multiple sequence alignments of selected miRNA families), in which the hairpin sequences are similar while the mature sequences differ greatly. The situation is reversed in the families that are merged in the miFam classification. One example is the mir-25, 92/mir-92b case, in which the mature sequences are almost identical while the rest of the sequences share little sequence similarity (See additional file 6). In only three cases, the families defined by PBC intersect with (overlap with but are not covered by) the families defined by miFam, but all these miRNAs are tandem clustered miRNAs which diverge from each other much more than most miRNA homologues.

Taken together, the comparison between the PBC and the miFam classifications showed that most of the families were consistent. Most of the differences stemmed likely from the differences in treating information from the mature sequences. While the PBC analysis pipeline relies totally on the precursor sequences, the miFam classification may have taken certain reference from the similarity between the mature sequences.

#### Classification of new miRNAs

While preparing the manuscript, a new release of miRBase sequences (Release 9.0) became available. We used our PBC analysis pipeline to classify the 12 new human miRNAs in this release by treating them as the confirmation set. The results showed that hsa-mir-758 can be assigned to the hsa-mir-379,380, 411 family; hsa-mir-767 can be assigned to the hsa-mir-105 family; and hsa-mir-802 can be assigned to the hsa-mir-511 family. The sequence alignments and the support levels among the trees can be found in additional file 7: Classification of new human miRNAs in Release 9.0. The hsa-mir-758 case is present in miFam already, while the other two cases have not been reported.

## Discussion

From an evolutionary point of view, miRNAs are a heterogeneous group of sequences. First, miRNAs are of heterogeneous evolutionary origins. Most of the miRNAs are not related to each other. They are categorized together as miRNAs just because they share certain common sequence features and functional mechanisms [6]. As is shown in the phylogenetic distribution of miRNA families in this study, miRNAs also differ greatly as to their levels of conservation across the species. Second, miRNAs differ in their evolutionary patterns. Some, the let-7 family for example, maintain almost identical mature forms in evolution. Others, the mir-10, 99, 100, 125 family for example, diverge in the mature forms (See additional file 8: The mir-10, 99, 100, 125 family).

The evolutionary distance between miRNAs is the underlying basis for the classification of miRNAs. The heterogeneous nature of miRNA sequences has made it impossible to use a single model to summarize the evolutionary distance between miRNAs. In context, many current methods actually manually inspect the classification process, which brings in a heavy human factor.

In the PBC analysis pipeline, we used a non-parametric approach. The analysis depended totally on the precursor sequences, and no model of the evolutionary distances between miRNAs was assumed *a priori*. The classification criteria were essentially derived from the data or were based on knowledge from the literature (the reference families). The human factor was minimized in the classification process. Meanwhile, the reliability of the families can be evaluated by their support levels in the bootstrap trees.

## Conclusion

The PBC analysis pipeline is an efficient method for classifying large numbers of heterogeneous miRNA sequences. The analysis pipeline assumes no models for the evolutionary distances between miRNAs. It requires minimum human involvement and provides a method to evaluate the reliability of the classification results. This analysis pipeline is efficient for classifying genome-wide sets of miRNA sequences. It is also an efficient method to classify newly cloned individual miRNAs into existing miRNA families.

## Methods

### Implementation

The miRNA sequences were retrieved from miRBase [10] (Release 8.2, Jul. 2006; Release 9.0, Oct. 2006). MiRNAs from six species, including *H. sapiens *(hsa, 462 entries), *M. musculus *(mmu, 358 entries), *G. gallus *(gga, 152 entries), *D. rerio *(dre, 337 entries), *D. melanogaster *(dme, 78 entries), and *C. elegans *(cel, 114 entries), were chosen for this study. miFam family information was retrieved from the same release.

Multiple sequence alignment (MSA) was carried out with CLUSTALW 1.83 [22]; neighbor-joining trees with bootstrap were inferred by Mega 3.1 [23] with 1000 bootstrap replications and p-distance substitution model. All the other data analysis was carried out by customized Perl modules and scripts, which were attached as the additional file 9: The software and instruction for the PBC analysis pipeline. The codes are also available at [25].

## Authors' contributions

XG and YH designed the study. YH carried out the data analysis. Both authors contributed to the manuscript writing. All authors read and approved the final manuscript.

## Supplementary Material

Additional File 1 Classification by BLAST. The classifications of miRNAs using different BLAST bit score cutoff values were compared to the miFam classification in the aspects of the cumulative miRNA difference and the composition of the families. Click here for file

Additional File 2 Best common ancestor (BCA). This file illustrates how the BCA is defined for a group of nodes. Click here for file

Additional File 3 Reference miRNA families. This file shows the composition of the reference families used in this analysis and the sequence identity range between miRNA precursors in the families. Click here for file

Additional File 4 Full list of the families with human member. The list of families from the classification of the miRNAs from six animal species, *H. sapiens, M. musculus, G. gallus, D. rerio, D. melanogaster*, and *C. elegans *using human miRNAs as the index. Only the families having human miRNAs are listed. Click here for file

Additional File 5 Supporting levels of the families. The supporting levels in the bootstrap trees of the human members in each family. Click here for file

Additional File 6 Multiple sequence alignments of selected miRNA families. The multiple sequence alignments of the miRNA families mentioned in the main text. Click here for file

Additional File 7 Classification of new human miRNAs in Release 9.0. The multiple sequence alignment and supporting levels (format see additional file 5) of the three families that three new human miRNAs in Release 9.0 have been assigned to. Click here for file

Additional File 8 The mir-10, 99, 100, 125 family. Sequence alignment and selected secondary structure of the miRNAs in the mir-10, 99, 100, 125 family. Click here for file

Additional File 9 The software and instruction for the PBC analysis pipeline. The PERL scripts of the programs used in the PBC analysis pipeline, and the instructions. The miRNAs sequences are retrieved from miRBase [10] (Release 8.2). Click here for file

## Figures and Tables

**Table 1:** Distribution of miRNA families in different categories of conservation

	**Hsa**	**Mmu**	**Gga**	**Dre**	**Dme**	**Cel**
***Category I***	47(15)	47(15)	41(13)	72(14)	22(15)	6(6)
***Category II***	146(83)	134(76)	106(62)	197(75)		
***Category III***	269(150)	177(137)			1(1)	7(7)
***Category IV***			5(3)	68(21)	55(38)	101(90)

* Each grid shows the number of miRNAs. The number in parentheses is the number of families.

**Table 2:** The human members of miRNA families in different conservation categories

	**Human miRNAs**
***Category I***	(let-7, mir-98), (mir-1, mir-206), (mir-10, mir-100, mir-125, mir-99), (mir-124), (mir-133), (mir-182), (mir-184), (mir-210), (mir-219), (mir-32), (mir-34), (mir-451), (mir-7), (mir-9), (mir-92)
***Category II***	(mir-101), (mir-103, mir-107), (mir-106, mir-17, mir-18, mir-20, mir-93), (mir-122), (mir-126), (mir-128), (mir-129), (mir-130, mir-301), (mir-132, mir-212), (mir-135), (mir-137), (mir-138), (mir-139), (mir-140), (mir-141, mir-200), (mir-142), (mir-143), (mir-144), (mir-145), (mir-146), (mir-147), (mir-148, mir-152), (mir-15), (mir-150), (mir-153), (mir-155), (mir-16, mir-195), (mir-181), (mir-183), (mir-187), (mir-19), (mir-190), (mir-191, mir-637), (mir-192), (mir-193), (mir-194), (mir-196), (mir-199), (mir-202), (mir-203), (mir-204, mir-211), (mir-205), (mir-208), (mir-21), (mir-214), (mir-215), (mir-216), (mir-217), (mir-218), (mir-22), (mir-220), (mir-221), (mir-222), (mir-223), (mir-23), (mir-24), (mir-25), (mir-26), (mir-27), (mir-29), (mir-30), (mir-302), (mir-31), (mir-33, mir-33), (mir-338), (mir-363), (mir-365), (mir-367), (mir-375), (mir-383), (mir-425), (mir-429), (mir-449), (mir-455), (mir-489), (mir-490), (mir-499), (mir-568, mir-620), (mir-585), (mir-590), (mir-639), (mir-92b), (mir-96)
***Category III***	(mir-105), (mir-127), (mir-134, mir-412), (mir-136), (mir-149), (mir-151, mir-28), (mir-154, mir-323, mir-329, mir-369, mir-377, mir-381, mir-382, mir-410, mir-453, mir-485, mir-487, mir-494, mir-495, mir-496, mir-539, mir-655, mir-656), (mir-185), (mir-186), (mir-188, mir-362, mir-500, mir-501, mir-502, mir-532, mir-660), (mir-197), (mir-198), (mir-224), (mir-296), (mir-299, mir-579), (mir-320), (mir-324, mir-544), (mir-325, mir-493), (mir-326), (mir-328, mir-483), (mir-330, mir-560), (mir-331), (mir-335), (mir-337), (mir-339), (mir-340), (mir-342, mir-610), (mir-345, mir-378), (mir-346), (mir-361), (mir-368, mir-376), (mir-370), (mir-371, mir-372, mir-512), (mir-373, mir-598), (mir-374, mir-542), (mir-379, mir-380, mir-411), (mir-384), (mir-409), (mir-421, mir-545, mir-95), (mir-422, mir-423), (mir-424), (mir-431), (mir-432), (mir-433), (mir-448), (mir-449b), (mir-450), (mir-452), (mir-484), (mir-486, mir-612), (mir-488), (mir-491), (mir-492), (mir-497, mir-600), (mir-498), (mir-503), (mir-504), (mir-505), (mir-506, mir-507, mir-508, mir-509, mir-510, mir-513, mir-514, mir-652), (mir-511), (mir-515, mir-516, mir-517, mir-518, mir-519, mir-520, mir-521, mir-522, mir-523, mir-524, mir-525, mir-526, mir-527), (mir-548, mir-570, mir-603), (mir-549), (mir-550), (mir-551), (mir-552), (mir-553, mir-626), (mir-554), (mir-555), (mir-556), (mir-557), (mir-558), (mir-559), (mir-561), (mir-562), (mir-563), (mir-564), (mir-565, mir-594), (mir-566), (mir-567), (mir-569), (mir-571), (mir-572, mir-638), (mir-573), (mir-574), (mir-575), (mir-576), (mir-577), (mir-578), (mir-580), (mir-581), (mir-582), (mir-583), (mir-584), (mir-586), (mir-587, mir-592), (mir-588), (mir-589), (mir-591), (mir-593), (mir-595), (mir-596, mir-650), (mir-597), (mir-599), (mir-601, mir-642), (mir-602), (mir-604), (mir-605), (mir-606), (mir-607), (mir-608), (mir-609), (mir-611), (mir-613), (mir-614), (mir-615), (mir-616), (mir-617), (mir-618), (mir-619), (mir-621, mir-662), (mir-622), (mir-623), (mir-624), (mir-625), (mir-627), (mir-628), (mir-629), (mir-630), (mir-631, mir-640), (mir-632, mir-661), (mir-633), (mir-634), (mir-635), (mir-636), (mir-641), (mir-643), (mir-644), (mir-645), (mir-646), (mir-647), (mir-648), (mir-649), (mir-651), (mir-653), (mir-654), (mir-657), (mir-658), (mir-659), (mir-663)

* Families are separated by parentheses, and the suffixes are ignored except for mir-92b and mir-449b.

**Table 3:** Comparison of the family composition between the PBC classification and the miFam classification

	***miRNAs***
***Families with more members by PBC***	(10a, 10b, 99a, 99b, 100, 125a, 125b-1, 125b-2, cel-lin-4), (21, mmu-468), (28, 151, mmu-708), (32, dme-mir-31a), (150, dre-150), (134, 412), (182, dme-263a, dme-263b), (188, 362, 500, 501, 502, 532, 660), (190, dre-190b), (191, 637), (197, mmu-705), (302a, 302b, 302c, 302d, dre-430b), (324, 544), (325, 493), (328, 483), (330, 560), (337, cel-241), (342, 610), (345, 378), (374, 542), (383, mmu-672), (422a, 423), (425, dre-731), (451, dme-14), (452, cel-358), (486, 612), (497, 600), (506, 507,508, 509, 510, 513-1, 513-2, 514-1, 514-2, 514-3, 652), (587, 592)
***Families with more members by miFam***	(15a, 15b, 16-1, 16-2, 195), (25, 92-1, 92-2, 92b), (29a, 29b-1, 29b-2, 29c, dme-285), (31, dme-31a, dme-31b), (33, dme-33), (141, 200a, 200b, 200c, 429), (192, 215), (221, 222), (424, mmu-322), (mmu-216a, mmu-216b)
***Families intersect between the two classifications***	(154, 323, 329-1, 329-2, 369, 377, 381, 382, 409, 410, 453, 485, 487a, 487b, 494, 495, 496, 539, 655, 656), (299, 548a-1,548a-2, 548a-3, 548b, 548c, 548d-1, 548d-2, 570, 579, 603), (371, 372, 512-1, 512-2, mmu-290, mmu-291a, mmu-291b, mmu-292, mmu-293, mmu-294, mmu-295)

* "hsa" and "mir" are omitted wherever no confusion can be caused. The miRNAs are human miRNAs if no prefixes are present.
