# Supplementary material for: A bootstrap based analysis pipeline for efficient classification of phylogenetically related animal miRNAs
Source: BMC Genomics. 2007 Mar 6;8:66. doi: 10.1186/1471-2164-8-66 (PMC1832191; doi:10.1186/1471-2164-8-66)
Supplement: Additional File 1 — Classification by BLAST. The classifications of miRNAs using different BLAST bit score cutoff values were compared to the miFam classification in the aspects of the cumulative miRNA difference and the composition of the families. [file 1471-2164-8-66-S1.doc]

**Classification by BLAST**

The precursor sequences of miRNAs from the six species were aligned (by BLAST, e=0.1 to include most of the hits) against each other. miRNA pairs with bit score above the bit score cutoff value where clustered together using a single linkage approach. We tested bit score cutoff values ranging from 20 to 40, bit by bit. The resulting families were compared to the miFam families from two aspects. First, we examined the cumulative miRNA differences between the classification by BLAST (at each cutoff) and the miFam. For a pair of comparable families, the miRNAs can be partitioned into three parts: A=(in the BLAST family only), B=(in the miFam family only) and C=(in both families). We defined the miRNA difference as the size of A minus the size of B. The cumulative miRNA difference is the sum over all the comparable families. The result is shown in the follow figure. The figure shows that the BLAST classifications of the families are very sensitive to the changes in the bit score cutoff, to the extent that a change of 1-2 bits can cause a vast change in the family compositions. It also shows that the cumulative miRNA difference between PBC and miFam is smaller than those by BLAST (54 for PBC, 170 for bit score cutoff 29 and 30, and -78 for bit score cutoff 31 and 32). Second, as the cumulative miRNA difference only examines the general difference in family sizes between different methods, we also examined the actual family composition of the well known families (let-7, mir-1, mir-10, mir-17 and mir-124) at bit cutoff values 30 and 31. At bit score cutoff 30, only the mir-124 family was identical to the miFam classification. Let-7 and mir-1 families were merged together. Mir-10 and mir-17 families were broken up into smaller groups. At bit score cutoff 31, let-7, mir-1 and mir-124 families were identical to the miFam classification. Mir-10 and mir-17 were still broken up. Besides these families, there was also a serious inconsistence in the rest families, including the segregation of many miRNAs with the same id numbers in different families. These results showed that a fixed bit score cutoff is inadequate for classifying the miRNA families.
