# Supplementary material for: A bootstrap based analysis pipeline for efficient classification of phylogenetically related animal miRNAs
Source: BMC Genomics. 2007 Mar 6;8:66. doi: 10.1186/1471-2164-8-66 (PMC1832191; doi:10.1186/1471-2164-8-66)

**The Best Common Ancestor (BCA)**

The BCA node for nodes A, B and C has the best bootstrap value among all the Common Ancestor (CA) nodes for A, B and C. The “Vote” algorithm decides the reliability of a family based on the boot strap value of the BCA of the family members in the bootstrap trees.


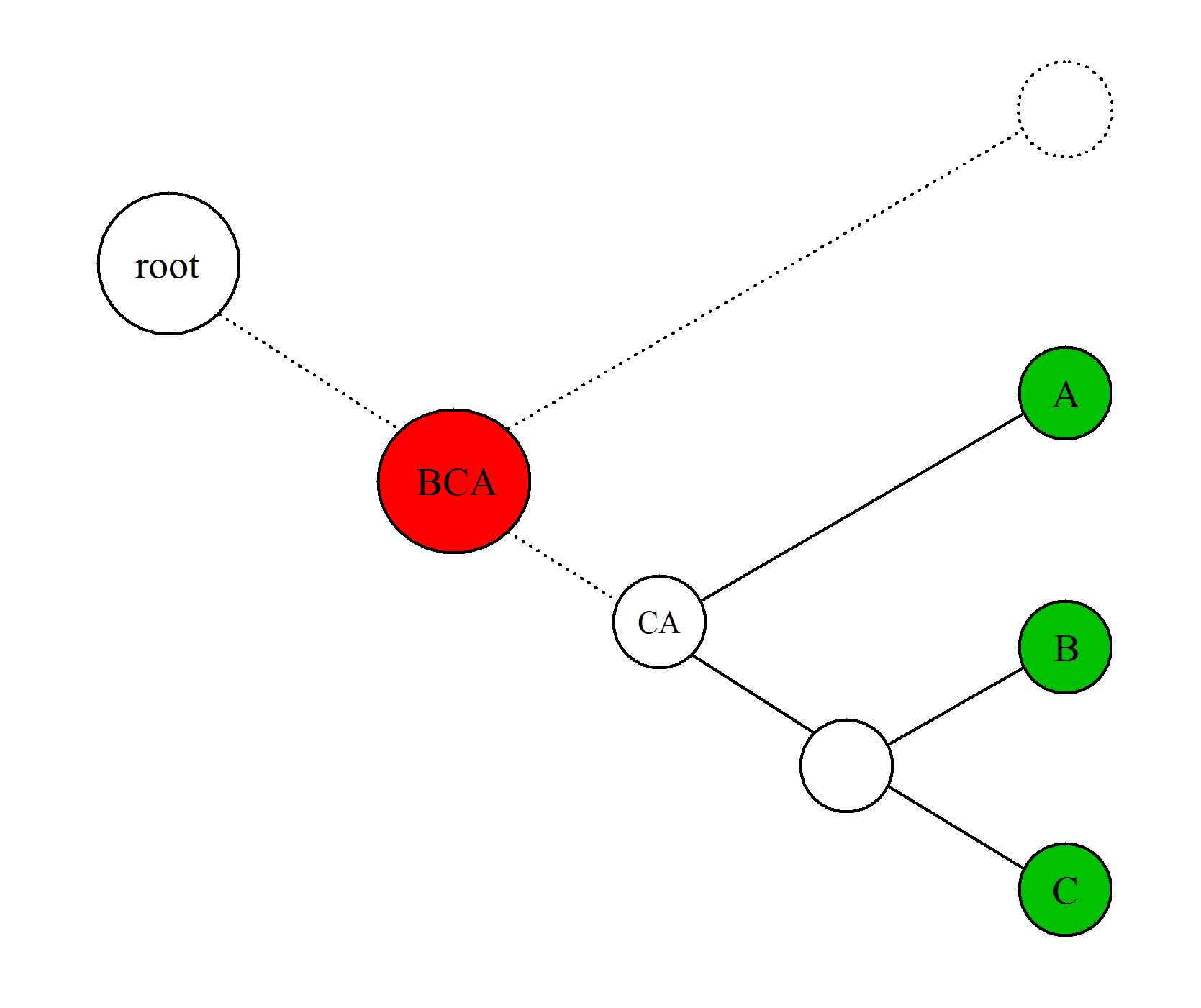

Supplement: Additional File 2 — Best common ancestor (BCA). This file illustrates how the BCA is defined for a group of nodes. [file 1471-2164-8-66-S2.doc]
