# Supplementary material for: A bootstrap based analysis pipeline for efficient classification of phylogenetically related animal miRNAs
Source: BMC Genomics. 2007 Mar 6;8:66. doi: 10.1186/1471-2164-8-66 (PMC1832191; doi:10.1186/1471-2164-8-66)
Supplement: Additional File 3 — Reference miRNA families. This file shows the composition of the reference families used in this analysis and the sequence identity range between miRNA precursors in the families. [file 1471-2164-8-66-S3.doc]

**Reference families and their sequence identity range**

> hsa-mir-17 hsa-mir-18a hsa-mir-18b hsa-mir-20a hsa-mir-20b hsa-mir-93 hsa-mir- hsa-mir-106a hsa-mir-106b

Sequence identity range in human: 0.588235294117647 - 0.887323943661972

Sequence identity range in all species: 0.579710144927536 - 0.985915492957746

> hsa-mir-1-1 hsa-mir-1-2 hsa-mir-206

Sequence identity range in human: 0.75 - 0.873015873015873

Sequence identity range in all species: 0.645833333333333 - 0.971830985915493

> hsa-mir-124a-1 hsa-mir-124a-2 hsa-mir-124a-3

Sequence identity range in human: 0.891566265060241 - 0.916666666666667

Sequence identity range in all species: 0.613861386138614 - 1

> hsa-let-7a-1 hsa-let-7a-2 hsa-let-7a-3 hsa-let-7b hsa-let-7c hsa-let-7d hsa-let-7e hsa-let-7f-1 hsa-let-7f-2 hsa-let-7g hsa-let-7i hsa-mir-98

Sequence identity range in human: 0.609756097560976 - 0.901234567901235

Sequence identity range in all species: 0.569620253164557 - 1
