# Supplementary material for: A bootstrap based analysis pipeline for efficient classification of phylogenetically related animal miRNAs
Source: BMC Genomics. 2007 Mar 6;8:66. doi: 10.1186/1471-2164-8-66 (PMC1832191; doi:10.1186/1471-2164-8-66)
Supplement: Additional File 6 — Multiple sequence alignments of selected miRNA families. The multiple sequence alignments of the miRNA families mentioned in the main text. [file 1471-2164-8-66-S6.doc]

**Multiple sequence alignment of several miRNA families discussed in the text.**

**1. MSA of mir-34 miRNAs**

MSA of the hairpin sequences

CLUSTAL W (1.83) multiple sequence alignment

cel-mir-34 ------CGGACAAUGCUCGAGAGGCAGUGUGGUUAGCUGGUUGCAU--AUU--------U

dme-mir-34 ----AAUUGGCUAUGCGCUUU-GGCAGUGUGGUUAGCUGGUUGUGU--AGC--------C

hsa-mir-34c ---------AGUCUAGUUACUAGGCAGUGUAGUUAGCUGAUUGCUA--AUA--------G

mmu-mir-34c ---------AGUCUAGUUACUAGGCAGUGUAGUUAGCUGAUUGCUA--AUA--------G

gga-mir-34c ---------AGCCUGGUUACCAGGCAGUGUAGUUAGCUGAUUGCCA--CCA--------G

dre-mir-34c ------UGCUGUGUGGUCACCAGGCAGUGCAGUUAGUUGAUUACAA--UCC--------A

gga-mir-34b --------GUGCUUGGUUUGCAGGCAGUGUAGUUAGCUGAUUGUACCCAGC--------G

dre-mir-34b --------GGGGUUGGUCUGUAGGCAGUGUUGUUAGCUGAUUGUUUCAUAU--------G

gga-mir-34a -GCCAGCUGUGAGUGUUUCUUUGGCAGUGUC-UUAGCUGGUUGUUGUGAGCAAUAGUUAA

mmu-mir-34a --CCAGCUGUGAGUAAUUCUUUGGCAGUGUC-UUAGCUGGUUGUUGUGAGUAUUAGCUAA

hsa-mir-34a GGCCAGCUGUGAGUGUUUCUUUGGCAGUGUC-UUAGCUGGUUGUUGUGAGCAAUAG-UAA

dre-mir-34 ---CUGCUGUGAGUGGUUCUCUGGCAGUGUC-UUAGCUGGUUGUUGUGUGGAGUGA-GAA

hsa-mir-34b --------GUGCUCGGUUUGUAGGCAGUGUCAUUAGCUGAUUGUACUGUG--------GU

mmu-mir-34b --------GUGCUCGGUUUGUAGGCAGUGUAAUUAGCUGAUUGUAGUGCG--------GU

******* **** ** **

cel-mir-34 CCUUGACAACGGCUACC-UUCACUGCCACCCCGAACAUGUCGUCCAUCUUUGAA

dme-mir-34 AAUUAUUGCCGUUGACAAUUCACAGCCACUAUCUUCACUGCCGCCGCGACAAGC

hsa-mir-34c UAC--CAAUCACUAACC--ACACGGCCAGGUAAAAAGAUU--------------

mmu-mir-34c UAC--CAAUCACUAACC--ACACAGCCAGGUAAAAAGACU--------------

gga-mir-34c GAC--CAAUCACUAACC--ACACAGCCAGGUAAAAAG-----------------

dre-mir-34c UAAAGUAAUCACUAACC--UCACUACCAGGUGAAGGCUAGUA------------

gga-mir-34b CCCCACAAUCACUAAAU--UCACUGCCAUCAAAACAAGGCAC------------

dre-mir-34b AACUAUAAUCACUAACC--AUACUGCCAACACAACAACCUACA-----------

gga-mir-34a GGAAGCAAUCAGCAAGU--AUACUGCCCUAGAAGUGCUACACAUUGUUGGGCC-

mmu-mir-34a GGAAGCAAUCAGCAAGU--AUACUGCCCUAGAAGUGCUGCACAUUGU-------

hsa-mir-34a GGAAGCAAUCAGCAAGU--AUACUGCCCUAGAAGUGCUGCACGUUGUGGGGCCC

dre-mir-34 CGAAGCAAUCAGCAAGU--AUACUGCCGCAGAAACUCGUCACCUU---------

hsa-mir-34b GGUUACAAUCACUAACU--CCACUGCCAUCAAAACAAGGCAC------------

mmu-mir-34b GCUGACAAUCACUAACU--CCACUGCCAUCAAAACAAGGCAC------------

* * ** **

MSA of the mature sequences

CLUSTAL W (1.83) multiple sequence alignment

dre-miR-34c -AGGCAGUGCAGUUAGUUGAUUAC-

mmu-miR-34c -AGGCAGUGUAGUUAGCUGAUUGC-

gga-miR-34c -AGGCAGUGUAGUUAGCUGAUUGC-

hsa-miR-34c -AGGCAGUGUAGUUAGCUGAUUGC-

gga-miR-34b CAGGCAGUGUAGUUAGCUGAUUG--

hsa-miR-34b UAGGCAGUGUCAUUAGCUGAUUG--

mmu-miR-34b UAGGCAGUGUAAUUAGCUGAUUG--

dre-miR-34b UAGGCAGUGUUGUUAGCUGAUUG--

gga-miR-34a -UGGCAGUGUC-UUAGCUGGUUGUU

hsa-miR-34a -UGGCAGUGUC-UUAGCUGGUUGUU

mmu-miR-34a -UGGCAGUGUC-UUAGCUGGUUGUU

dre-miR-34 -UGGCAGUGUC-UUAGCUGGUUGU-

dme-miR-34 -UGGCAGUGUGGUUAGCUGGUUG--

cel-miR-34 -AGGCAGUGUGGUUAGCUGGUUG--

******* **** ** **

**2. MSA of mir-134 and mir-142**

MSA of the hairpin sequence

CLUSTAL W (1.83) multiple sequence alignment

hsa-mir-412 CUGGGGUACGGGGAUGGAUGGUCGACCAGUUGGAAA-GUAAU-UGUUUCUAAUGUACUUC

mmu-mir-412 ---GGGUAUGGG-ACGGAUGGUCGACCAGCUGGAAA-GUAAU-UGUUUCUAAUGUACUUC

hsa-mir-134 -CAGGGUGUG----UGACUGGUUGACCAGAGGGGCAUGCACUGUGUUCACCCUGUGGGCC

mmu-mir-134 --AGGGUGUG----UGACUGGUUGACCAGAGGGGCGUGCACUCUGUUCACCCUGUGGGCC

**** * * **** ****** ** * * * **** *** *

hsa-mir-412 ACCUGGUCCACUAGCCGUCCGUAUCCGCUGCAG

mmu-mir-412 ACCUGGUCCACUAGCCGUCGGUGCCC-------

hsa-mir-134 ACCUAGUC-ACCAACCCUC--------------

mmu-mir-134 ACCUAGUC-ACCAACCCU---------------

**** *** ** * ** *

MSA of the mature sequence

CLUSTAL W (1.83) multiple sequence alignment

hsa-miR-412 ACUUCACCUGGUCCACUAGCCGU-

mmu-miR-412 ACUUCACCUGGUCCACUAGCCGU-

hsa-miR-134 --UGUGACUGGUUGACCAGAGGG-

mmu-miR-134 --UGUGACUGGUUGACCAGAGGGG

* ***** ** ** *

**3. MSA of mir-25, mir-92 and mir-92b**

MSA of the hairpin sequences.

CLUSTAL W (1.83) multiple sequence alignment

dre-mir-92b ------UCCUACGGGCAGGGAGGUGUGGGAUGUUGUGCAGUGUUGUUCAAUCUCC---CG

hsa-mir-92b CGGGCCCCGGGCGGGC-GGGAGGGACGGGACGCGGUGCAGUGUUGUUUUUUCCCC---CG

dme-mir-92a -----AAUAUGAAUUUCCCGUAGGACGGGAAGGUGU-CAACGUU-UUGCAUUUCG---AA

dme-mir-92b ----UAAAACGUCACCUGAUGUAGGCCGUGCCCAGUGCUUAUUUGUUGCAUUUUCGA-AA

hsa-mir-92-1 ------------CUUUCUACACAGGUUGGGAUCGGU--UGCAAUGCUGUGUUUCU---GU

mmu-mir-92-1 ------------CUUUCUACACAGGUUGGGAUUUGU--CGCAAUGCUGUGUUUCUCU-GU

gga-mir-92 ------------CUUUCUACACAGGUUGGGAUCAGU--UGCAAUGCUGUGCGUUUC---U

dre-mir-92a-1 ------UGGUCCCUUUCUGCGCAGGUUGGGAUUGGU--AGCAAUGCUGUGUGUUUU---G

dre-mir-92a-2 ---ACAGCAUCCCUUUCUUUGCAGGUUGGGAUCGGC--CGCAAUGCUCUGUGC--U---G

hsa-mir-92-2 --------------UCAUCCCUGGGUGGGGAUUUGU--UGCAUUACU-UGUGUUCUAUAU

mmu-mir-92-2 -------UGCCCAUUCAUCCACAGGUGGGGAUUGGU--GGCAUUACU-UGUGUUAGAUAU

hsa-mir-25 ---------GGCCAGUGUUGAGAGGCGGAGACUUGG--GCAAUUGCUGGACGCUGC--CC

mmu-mir-25 ---------GGCCAGUGUUGAGAGGCGGAGACUUGG--GCAAUUGCUGGACGCUGC--CC

dre-mir-25 ----------GCCGGCGCUGAGAGGCGGAGACUUGG--GCAGCUGCCGUCAUUCCC--AG

* * *

dre-mir-92b CCAAUAUUGCACUCGUCCCGGCCUCCCU-GAC--------------

hsa-mir-92b CCAAUAUUGCACUCGUCCCGGCCUCCGG-CCCCCCCGGCCC-----

dme-mir-92a UAAACAUUGCACUUGUCCCGGCCUAUGG-GCGGUUUGUAAUAAACA

dme-mir-92b UACAAAUUGCACUAGUCCCGGCCUGCAAUGAGUGUCGCAGUCGAC-

hsa-mir-92-1 AUGGUAUUGCACUUGUCCCGGCCUGUUGAGUUUGG-----------

mmu-mir-92-1 AUGGUAUUGCACUUGUCCCGGCCUGUUGAGUUUGG-----------

gga-mir-92 GUGGUAUUGCACUUGUCCCGGCCUGUUGAGGUUGG-----------

dre-mir-92a-1 AAGGUAUUGCACUUGUCCCGGCCUGUAAAGGAU-UGU---------

dre-mir-92a-2 GAAGUAUUGCACUUGUCCCGGCCUGUGAAGAGCAUGGGAAAUUGU-

hsa-mir-92-2 AAAGUAUUGCACUUGUCCCGGCCUGUGGAAGA--------------

mmu-mir-92-2 AAAGUAUUGCACUUGUCCCGGCCUGAGGAAGAAAGAGGGUU-----

hsa-mir-25 UGGGCAUUGCACUUGUCUCGGUCUGACAGUGCCGGCC---------

mmu-mir-25 UGGGCAUUGCACUUGUCUCGGUCUGACAGUGCCGGCC---------

dre-mir-25 AAGGCAUUGCACUUGUCUCGGUCUGACAGUGGCGGC----------

******** *** *** **

MSA of the mature sequences.

CLUSTAL W (1.83) multiple sequence alignment

dre-miR-92a UAUUGCACUUGUCCCGGCCUGU

gga-miR-92 UAUUGCACUUGUCCCGGCCUG-

mmu-miR-92 UAUUGCACUUGUCCCGGCCUG-

hsa-miR-92 UAUUGCACUUGUCCCGGCCUG-

dre-miR-92b UAUUGCACUCGUCCCGGCCUCC

hsa-miR-92b UAUUGCACUCGUCCCGGCCUC-

dme-miR-92b AAUUGCACUAGUCCCGGCCUGC

dme-miR-92a CAUUGCACUUGUCCCGGCCUAU

dre-miR-25 CAUUGCACUUGUCUCGGUCUGA

hsa-miR-25 CAUUGCACUUGUCUCGGUCUGA

mmu-miR-25 CAUUGCACUUGUCUCGGUCUGA

******** *** *** **
