# Supplementary material for: A bootstrap based analysis pipeline for efficient classification of phylogenetically related animal miRNAs
Source: BMC Genomics. 2007 Mar 6;8:66. doi: 10.1186/1471-2164-8-66 (PMC1832191; doi:10.1186/1471-2164-8-66)
Supplement: Additional File 7 — Classification of new human miRNAs in Release 9.0. The multiple sequence alignment and supporting levels (format see additional file 5) of the three families that three new human miRNAs in Release 9.0 have been assigned to. [file 1471-2164-8-66-S7.doc]

**Classification of new human miRNAs from release 9.0**

1)hsa-mir-758 hsa-mir-379 hsa-mir-380 hsa-mir-411

Alignment:

hsa-mir-411 -UGGUACUUGGAGAGAUAGUAGACCGUAUAGCGUACGCUUUAU---CUGUGACGUAUGUA

hsa-mir-379 -----------AGAGAUGGUAGACUAUGGAACGUAGGCGUUAUGAUUUCUGACCUAUGUA

hsa-mir-380 ------------AAGAUGGUUGACCAUAGAACAUGCGCUAUCU---CUGUGUCGUAUGUA

hsa-mir-758 GCCUGGAUACAUGAGAUGGUUGACCAGAGAGCACACGCUUUAU---UUGUGCCGUUUGUG

**** ** *** * * ** * * * ** * * ***

hsa-mir-411 ACACGGUCCACUAACCCUCAGUAUCAAAUCCAUCCCCGAG

hsa-mir-379 ACAUGGUCCACUAACUCU----------------------

hsa-mir-380 AUAUGGUCCAC--AUCUU----------------------

hsa-mir-758 ACCUGGUCCACUAACCCUCAGUAUCUAAUGC---------

* ******* * *

Support levels:

hsa 742 0.98

hsa_mmu 1347 0.98

hsa_gga 311 0.95

hsa_dre 157 0.95

hsa_cel 212 0.96

hsa_dme 1096 0.98

2) hsa-mir-767 hsa-mir-105-1 hsa-mir-105-2

Alignment:

hsa-mir-105-1 ---UGUGCAUCGUGGUCAAAUGCUCAGACUCCUGUGGUGGCUGCUCAUGCACCACGGAUG

hsa-mir-105-2 ---UGUGCAUCGUGGUCAAAUGCUCAGACUCCUGUGGUGGCUGCUUAUGCACCACGGAUG

hsa-mir-767 GCUUUUAUAUUGUAGGUUUUUGCUCAUGCACCAUGGUUGUCUGAGCAUGCAGCAUGCUUG

* * ** ** * ****** * ** * ** *** ***** ** * **

hsa-mir-105-1 UUUGAGCAUGUGCUACGGUGUCUA-------------------------

hsa-mir-105-2 UUUGAGCAUGUGCUAUGGUGUCUA-------------------------

hsa-mir-767 UCUGCUCAUACCCCAUGGUUUCUGAGCAGGAACCUUCAUUGUCUACUGC

* ** *** * * *** ***

Support levels:

hsa 883 0.95

hsa_mmu -1 -1

hsa_gga 805 0.97

hsa_dre 1332 0.98

hsa_cel 1064 0.96

hsa_dme 515 0.95

3) hsa-mir-802 hsa-mir-511-1 hsa-mir-511-2

Alignment:

hsa-mir-511-1 CAAUAGACACCCAUCGUGUCUUUUGCUCUGCAGUCAGUAA-----AUAUUUUUUUGUGAA

hsa-mir-511-2 CAAUAGACACCCAUCGUGUCUUUUGCUCUGCAGUCAGUAA-----AUAUUUUUUUGUGAA

hsa-mir-802 -----------------GUUCUGUUAUUUGCAGUCAGUAACAAAGAUUCAUCCUUGUGUC

** * * * ************ ** * *****

hsa-mir-511-1 UGUGUAGCAAAAGACAGAAUGGUGGUCCAUUG-------------------

hsa-mir-511-2 UGUGUAGCAAAAGACAGAAUGGUGGUCCAUUG-------------------

hsa-mir-802 CAUCAUGCAACAAGGAGAAUCUUUGUCACUUAGUGUAAUUAAUAGCUGGAC

* **** * ***** * *** **

Support levels:

hsa 497 0.87

hsa_mmu 567 0.89

hsa_gga 415 0.97

hsa_dre 761 0.00

hsa_cel 838 0.91

hsa_dme 1036 0.90
