# Supplementary figures and images for: A bootstrap based analysis pipeline for efficient classification of phylogenetically related animal miRNAs
Source: BMC Genomics. 2007 Mar 6;8:66. doi: 10.1186/1471-2164-8-66 (PMC1832191; doi:10.1186/1471-2164-8-66)

**The mir-10,99,100,125 family.**


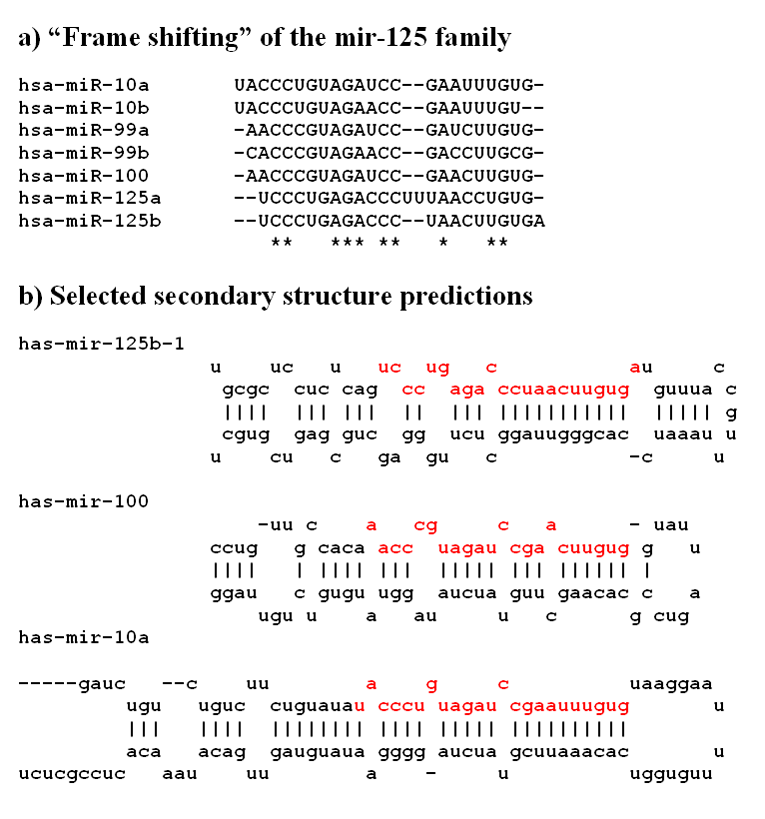

Supplement: Additional File 8 — The mir-10, 99, 100, 125 family. Sequence alignment and selected secondary structure of the miRNAs in the mir-10, 99, 100, 125 family. [file 1471-2164-8-66-S8.doc]
