# Supplementary material for: A bootstrap based analysis pipeline for efficient classification of phylogenetically related animal miRNAs
Source: BMC Genomics. 2007 Mar 6;8:66. doi: 10.1186/1471-2164-8-66 (PMC1832191; doi:10.1186/1471-2164-8-66)
Supplement: Additional File 9 — The software and instruction for the PBC analysis pipeline. The PERL scripts of the programs used in the PBC analysis pipeline, and the instructions. The miRNAs sequences are retrieved from miRBase [10] (Release 8.2). [file 1471-2164-8-66-S9.zip › ReadMeFirst.doc]

********************************************************************

*	ReadMeFirst for running the PBC software	*

********************************************************************

Synopsis:

This is an instruction for running the PBC software for the test case of miRNAs from the six species.

Test environment:

The Perl scripts have been tested on a PC running Fedora release 4. The programs require Perl version 5.6 and above. It also need the following packages: Storable, Data::Dumper, and Statistics::Descriptive. All these packages can be downloaded from the CPAN website. It is also recommended that Bioperl of version 1.4 and above be installed.

Directory Contents:

root:

ReadMeFirst.txt - instructions for using the software

test_storable.pl - a test tool for viewing Stroable files.

(We use the Storable module for storing temporary files)

Tools/miRNA/: modules used in the programs

treeNode.pm	- the definition of a treeNode object

treeUtils.pm	- methods for traverse the tree

RelMethods.pm	- Methods for dealing with relations

PhyloMethods.pm - Methods for miRNA data treatments

release82/: data files from miRBase release 8.2

hairpin.fa - miRNA precursor sequences

mature.fa - miRNA mature sequences

firstround/: data, programs and results for the first round of PBC

*.nwk	- the input bootstrap trees

batch_run.pl - the script for executing the programs for the

first round of PBC (see the script for details)

*.fa	- the correspondent fasta files for the nwk files

(* see Note 1)

get_mirna_sequences.pl - the program to generate the fasta files

secondround/: data, programs and results for the second round of PBC

*.nwk	- the input bootstrap trees

batch_run2.pl -the script for executing the programs for the

second round of PBC (see the script for details)

*.fa	- the correspondent fasta files for the nwk files

rel_get_sequences.pl - the program to generate the fasta files

cfm_set*.store	- the sequences to be confirmed, generated by

rel_get_sequences.pl. The confirmation set is the

combination of all these cfm_set*.store files.

PBC_fulllist.txt - the resulting full list of miRNA families

(only the families with human members)

PBC_human_mirna_category.txt - list and summary of huma families

of different levels of conservation

USAGE

1) Read the ReadMeFirst.txt file. Ensure your test platform is properly setup.

2) Go to directory "firstround", and execute batch_run.pl ("perl batch_run.pl"). The program will take around half an hour on a typical PC.

3) Go to directory "secondround", and execute batch_run2.pl ("perl batch_run2.pl"). The program will take around 1.5 hours on a typical PC. The overall results are PBC_fulllist.txt and PBC_human_mirna_category.txt.

Note

1) We provided the nwk files as the input of the analysis pipeline. The fasta files and the programs to generate the fasta files are also included in the directories. Third party softwares, Clustalw and Mega3.1,are needed to perform the multiple sequence alignment, phylogenetic tree building and bootstrapping to generate the nwk files from the fasta files.
